# Translation and validation of the Myasthenia Gravis Activities of Daily Living Questionnaire: Latvian version

**DOI:** 10.3389/fneur.2024.1397603

**Published:** 2024-05-27

**Authors:** Arta Grosmane, Ieva Roze, Marija Roddate, Gundega Ķauķe, Violeta Žukova, Ieva Glāzere, Maksims Zolovs, Viktorija Ķēniņa

**Affiliations:** ^1^Department of Neurology, Pauls Stradiņš Clinical University Hospital, Riga, Latvia; ^2^Department of Residency, Rīga Stradiņš University, Riga, Latvia; ^3^Department of Biology and Microbiology, Rīga Stradiņš University, Riga, Latvia; ^4^Statistics Unit, Rīga Stradiņš University, Riga, Latvia; ^5^Institute of Life Sciences and Technology, Daugavpils University, Daugavpils, Latvia; ^6^Institute of Oncology and Molecular Genetics, Rīga Stradiņš University, Riga, Latvia

**Keywords:** myasthenia gravis, MG-ADL, daily activities, scale, validation

## Abstract

**Introduction:**

Our aim was to translate, adapt and validate the Myasthenia Gravis Activities of Daily Living scale into the Latvian language and to evaluate this instrument (MG-ADL-L) in terms of construct validity and reliability.

**Methods:**

We enrolled patients with a confirmed MG diagnosis, who could speak Latvian fluently. We performed translation and adaptation according to the cross-cultural adaptation guidelines for self-reported measures. The patients were evaluated by a physician according to the Myasthenia Gravis Foundation of America classification (MGFA) and using the Myasthenia Gravis Composite Score (MGCS). Patients were asked to complete the MG-ADL-L and the 15-item Myasthenia Gravis Quality of Life (MGQOL15) Internal consistency was evaluated based on Cronbach’s α, reproducibility—Cohen’s weighted kappa and construct validity—Spearman’s correlation between the MG-ADL-L and the MGQOL15 and MGCS. We used the Kruskal–Wallis H test to compare the MG-ADL-L score distribution between the MGFA groups.

**Results:**

38 enrolled patients in the study. There was an acceptable internal consistency (Cronbach’s α = 0.76) and moderate to very good agreement between the test and retest scores (Cohen’s weighted kappa = 0.54 and 0.81). The MG-ADL-L showed a moderate positive correlation with the MGQOL15 (r = 0.5, *p* = 0.001) and the MGCS (r = 0.62, *p* < 0.001). There was a significant difference in MG-ADL-L scores between the MGFA groups (*p* = 0.007).

**Discussion:**

The MG-ADL-L is a valid and reliable self-reported scale to assess and evaluate symptom severity and the impact of the disease on the lives of patients with MG.

## 1 Introduction

Myasthenia gravis (MG) is a chronic autoimmune disorder in which the major symptoms are voluntary muscle weakness and fatigue, which worsens with repeated and prolonged use of muscles and can also fluctuate in severity (1). In addition to these symptoms, patients with MG show worse physical functioning and emotional well-being compared with the general population, as well as increased difficulty in activities of daily living (2).

The Myasthenia Gravis Activities of Daily Living (MG-ADL) scale was developed to assess symptom severity and the activity status in patients with MG and to evaluate the impact of the disease on the daily lives of these patients. It consists of eight items that assess impairment in the main MG-affected functions: bulbar, respiratory, limb and ocular. Each item is given scored from 0 to 3, with a maximum total score of 24. A higher score indicates greater severity (3). The MG-ADL can be self-reported or administered by a physician, as it has been shown that the results are concordant (4). It is one of the most commonly used outcome measures in clinical trials and it has strong correlation with the Quantitative Myasthenia Gravis (QMG) score, the Myasthenia Gravis Composite Score (MGCS) and the Myasthenia Gravis 15-Item Quality of Life scale (MG-QOL15) (3, 5). The MG-ADL is responsive to clinical improvement, and it can be also used to assess the progression of the disease and to determine the effectiveness of the treatment (6–8). This scale has been translated into other languages, and those versions have been validated (9–12). Due to the fact that there is no MG-specific assessment tool for the severity of symptoms and their effect on daily activities in the Latvian language, our aim was to translate and adapt the MG-ADL to Latvian and to evaluate the Latvian version (MG-ADL-L) in terms of construct validity and reliability.

## 2 Materials and methods

### 2.1 Patients

Patients were enrolled for study at Pauls Stradins Clinical University Hospital’s Centre for Rare Neurological Diseases. They were participating in a parallel study to determine the MG protein biomarker profile; this study used the same instruments. Several of these patients also participated in the MG-QOL15-L adaptation and validation research. Ethical approval was obtained from Riga Stradiņš University Ethics Committee. Informed consent was obtained from each participant.

The study included 38 patients who were ≥ 18 years old, of each gender and with a confirmed MG diagnosis (regardless of the type). Patients with severe MG—Myasthenia Gravis Foundation of America (MGFA) class V—or patients who did not speak Latvian were excluded from the study.

A sample size within the range of 30–50 was deemed sufficient, as recommended by established research on the determination of the sample size of reliability analysis (13, 14). Such sample size ensures a power of 80%, allowing to detect a moderate to high level of reliability with confidence.

### 2.2 Translation and adaptation

Translation and adaptation were performed according to the cross-cultural adaptation guidelines for self-reported measures (15). A neurologist and a professional translator with no medical background translated the MG-ADL to Latvian separately, creating two Latvian versions. Both translations were reviewed and combined by another researcher into the MG-ADL-L; there were no notable discrepancies. The developed MG-ADL-L was back translated and compared with the original English version. Afterwards, the MG-ADL-L was accepted.

The approved MG-ADL-L was tested by recruiting 10 patients who completed the scale in the waiting room before their outpatient visit. The patients were asked how well they comprehended and accepted the scale. After pilot test, no changes to the MG-ADL-L were deemed necessary.

### 2.3 Data collection

The 38 recruited participants were asked to complete the MG-ADL-L and the MG-QOL15-L in the waiting room before their visit with the neurologist. During the visit, the physician evaluated each participant based on the MGCS and MGFA classification. For all of the mentioned outcome measures, higher scores indicate greater symptom severity.

To test reproducibility, 20 participants were asked to complete the questionnaire at home 1 week later and to send in their results electronically to the nurse coordinator, who anonymised the data for the researchers. The patients were instructed to inform the nurse coordinator if they experienced a change or exacerbation in their symptoms during this week.

### 2.4 Statistical analysis

Patient demographics were analysed with descriptive statistics. IBM SPSS Statistics was used for the statistical analysis.

Reliability was evaluated based on Cronbach’s alpha, inter-item correlations and corrected item-total correlations. Cronbach’s α > 0.7 was deemed acceptable (16).

Cohen’s weighted kappa was used to assess reproducibility based on the test and retest scores. Cohen’s weighted kappa was interpreted as follows: > 0.41, moderate agreement; > 0.61, substantial agreement; and > 0.81, near perfect agreement.

To evaluate validity, Spearman’s correlation was used to determine the association between the MG-ADL-L and the MGQOL15, MG-ADL-L and MGCS. The Kruskal–Wallis H test was used to assess differences in the MG-ADL-L scores between the different MG severity groups (based on the MGFA classification).

## 3 Results

### 3.1 Participant characteristics

The mean age of the 38 included participants was 52.1 ± 14.4 years (95% CI: 47.4 to 56.8 years) (Table 1). There were 14 (37%) men and 24 (63%) women. The mean disease duration was 9.1 ± 1.4 years (range: 5 months to 34.8 years; 95% CI: 6.3 to 12.0 years). Of the 38 participants, 6 (16%) were at the first visit and 32 (84%) were at follow-up visits. According to the MGFA classification, 5 (13%) patients were MGFA I, 12 (32%) patients were MGFA IIa, 5 (13%) patients were MGFA IIb and 3 (8%) patients were MGFA IIIb. According to the MGFA post-intervention status classification (MGFA PIS), 13 of 38 patients (34%) reached either complete stable or pharmacological remission.

**Table 1:** Patient demographics.

Mean age (± SD)	52.1 (±14.4)				
Sex, male (%)/female (%)	14 (37%) / 24 (63%)				
Mean disease duration, years (± SD)	9.1 (±1.4)				
Confirmed thymoma in patient history (%)	6 (16%)				
Patient status according to MGFA and MGFA PIS classification (%)	**MGFA I**	**MGFA IIa**	**MGFA IIb**	**MGFA IIIb**	**CSR or PR**
	5 (13%)	12 (32%)	5 (13%)	3 (8%)	13 (34%)
Serological classification (%)	**AChR +**	**MuSK +**		**Seronegative**	
	34 (89%)	1 (3%)		3 (8%)	

AChR, acetylcholine receptor; CSR, complete stable remission; MGFA, Myasthenia Gravis Foundation of America; MGFA PIS, Myasthenia Gravis Foundation of America post-intervention status; MuSK, muscle-specific kinase; PR, pharmacological remission; SD, standard deviation.

Most of the patients (n = 34, 89%) were positive for acetylcholine receptor antibodies, while only 1 patient (3%) was positive for muscle-specific kinase antibodies. Three patients (8%) were seronegative. Out of the study sample, 6 patients (16%) had confirmed thymoma and all had undergone thymectomy previously.

Most of the patients (n = 9, 24%) were on triple therapy (pyridostigmine, a corticosterodid (i.e., prednisolone) and an immunosuppressant (i.e., azathioprine)). Seven patients (18%) were on pyridostigmine only, one (3%) patient received only corticosteroid, three (8%) patients used only an immunosuppressant. Three (8%) patients used pyridostigmine in combination with corticosteroid, 5 (13%) patients received pyridostigmine combined with immunosuppresant. Eight patients (21%) used corticosteroid combined with immunosuppressant. Two (5%) patients did not use any medication at all.

One patient (3%) had listed rheumatoid arthritis as a comorbidity (MG-ADL result—1), while 3 patients (8%) reported autoimmune thyroiditis MG-ADL results in a range from 1 to 3.

### 3.2 Translation

We successfully translated the MG-ADL into the Latvian language to generate the MG-ADL-L. There were no disagreements or discrepancies during the translation process. The patients were able to answer the questionnaire without any problems. There were no uncertainties or questions about the questionnaire during cognitive debriefing and data collection.

### 3.3 Reliability

The MG-ADL-L has acceptable internal consistency (Cronbach’s α = 0.76). Each item of the MG-ADL-L scale showed inter-item correlation ranging from 0.30 to 0.80 (Table 2). The mean inter-item correlation was 0.53. The double vision and eyelid droop items had the lowest corrected item-total correlation. Deletion of each item increased the internal consistency (Cronbach’s α = 0.79).

**Table 2:** Reliability and agreement between the test and retest scores of the Latvian version of the Myasthenia Gravis Activities of Daily Living (MG-ADL-L).

MG-ADL-L items	Mean ± standard deviation	Corrected item-total correlation	Cronbach’s α if the item was deleted	Test–retest Cohen’s weighted kappa
Talking	0.18 ± 0.46	0.40	0.75	1.00
Chewing	0.21 ± 0.41	0.72	0.71	0.83
Swallowing	0.18 ± 46	0.49	0.74	1.00
Breathing	0.37 ± 0.59	0.68	0.70	0.75
Brushing teeth or hair	0.18 ± 0.51	0.80	0.69	0.86
Arising from chair	0.26 ± 0.55	0.54	0.72	0.54
Double vision	0.79 ± 1.02	0.30	0.80	0.89
Eyelid droop	0.53 ± 0.95	0.30	0.79	0.75

### 3.4 Reproducibility

No patients reported exacerbation in their symptoms between the test and retest. The MG-ADL-L showed moderate to very good agreement between the test and retest scores. We noted the lowest agreement for the chair item (Cohen’s weighted kappa = 0.54, *p* = 0.005), while the talking, chewing, swallowing, brushing teeth or hair, and double vision items showed near perfect agreement (Cohen’s weighted kappa >0.81, *p* < 0.001). The eyelid droop and breathing items showed substantial agreement between the test and retest scores (Cohen’s weighted kappa = 0.75 for each).

### 3.5 Construct validity

**Figure 1:**
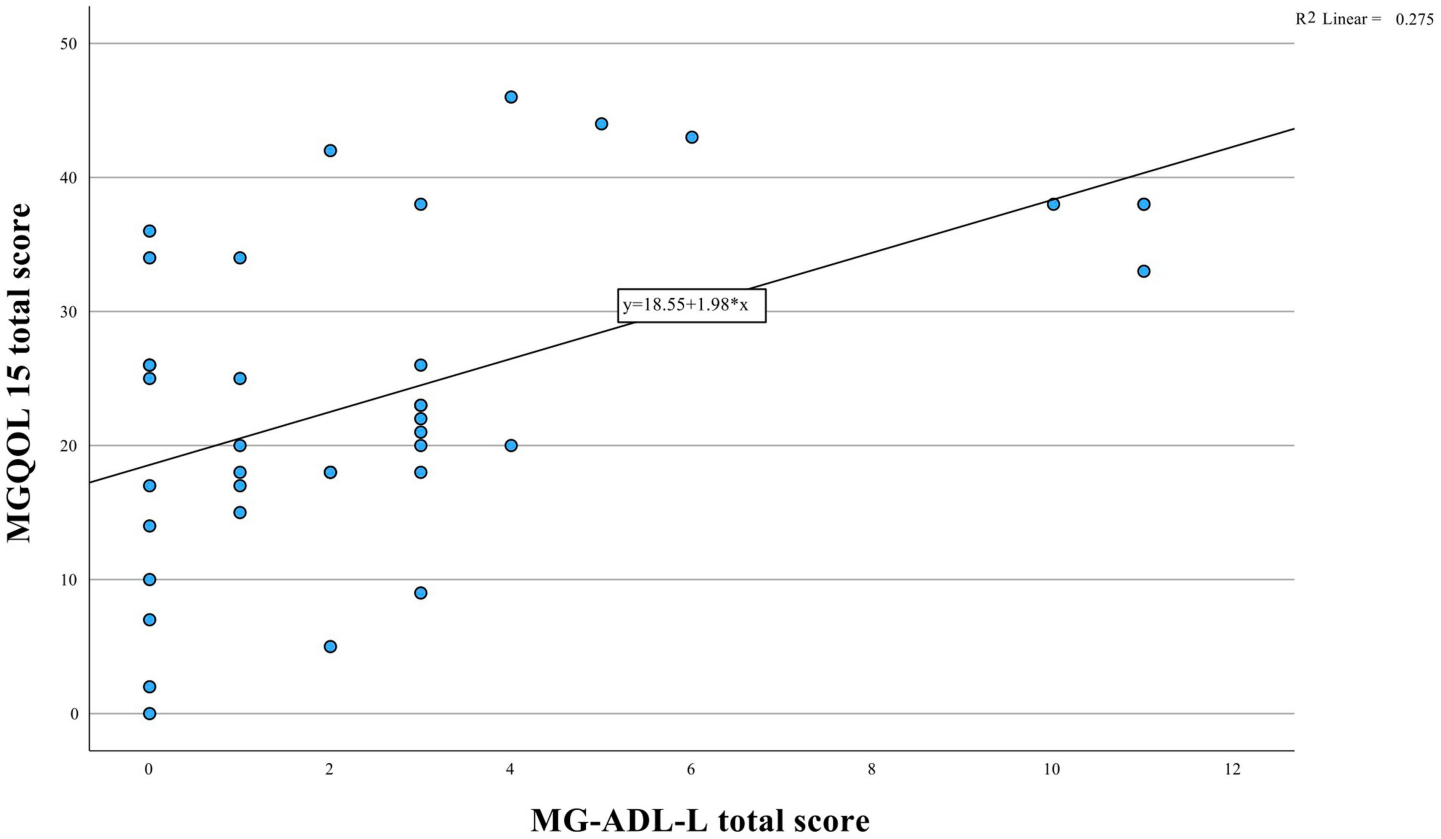
Relationship between MGQOL15 and MG-ADL-L instruments depicted. Statistical analysis reveals a significant positive correlation (*r* = 0.50, *p* = 0.001).

**Figure 2:**
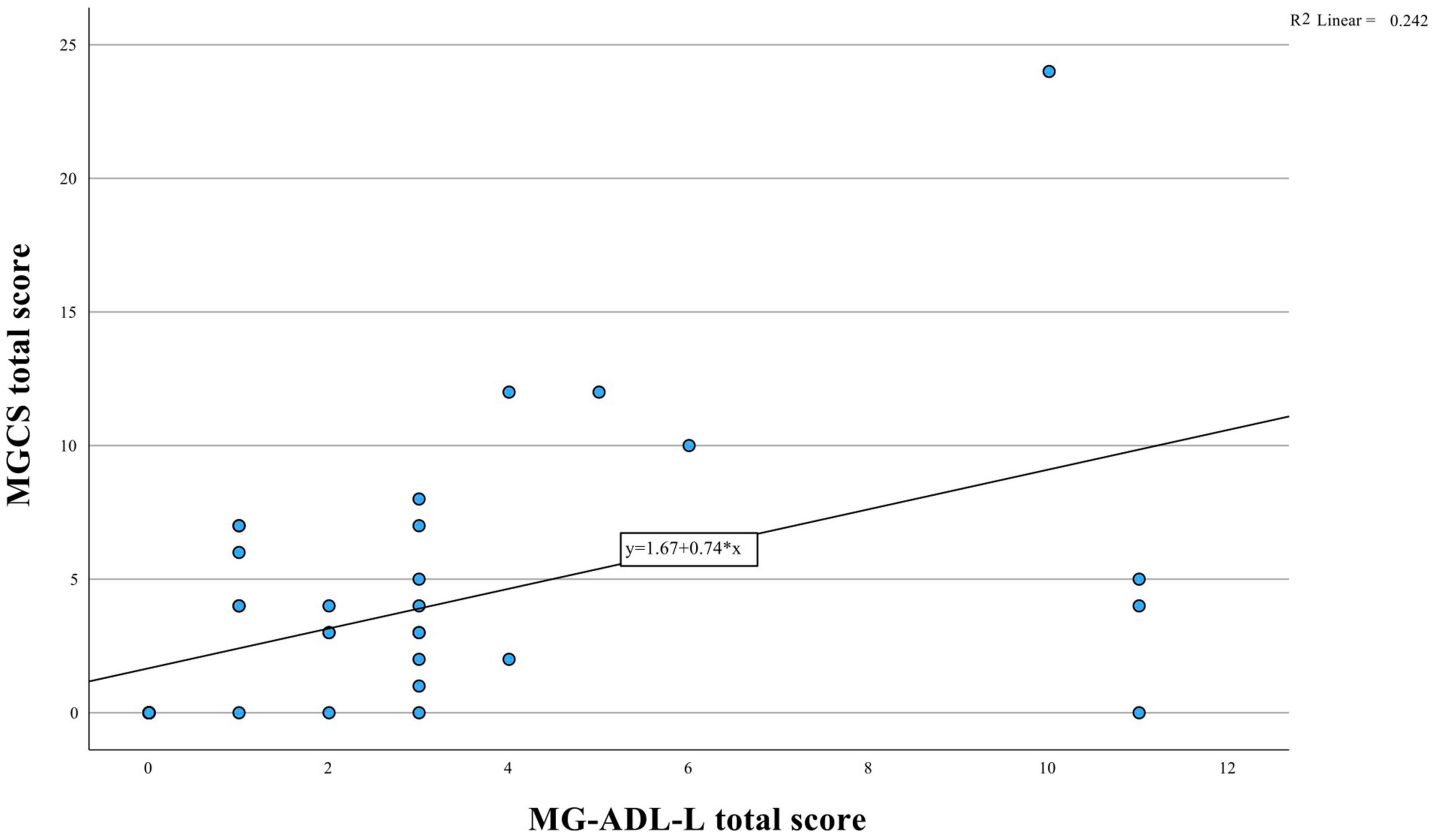
Analysis of MG-ADL-L and MGCS correlation illustrated in the figure. The data suggests a significant positive relationship (*r* = 0.62, *p* < 0.001).

The median MG-ADL-L, MGQOL15 and MGCS scores were 2.0 (interquartile range [IQR] = 3), 22.5 (IQR = 18) and 3.0 (IQR = 5), respectively. There was a moderate correlation between the MG-ADL-L and the MGQOL15 (Spearman’s correlation, r = 0.50, *p* = 0.001, Figure 1) and between the MG-ADL-L and the MGCS (r = 0.62, *p* < 0.001, Figure 2).

We found a significant difference in the MG-ADL-L scores across the MGFA groups (H = 14.061, *p* = 0.007; Table 3). There was no significant difference between the MGFA I and MGFA IIB subgroups (median = 2.0 for each subgroup; first to third quartile of 0.5–7.0 and 1.0–7.5, respectively; *p* = 1.000). Patients classified as MGFA IIIB showed higher MG-ADL-L scores (median = 5.0, first to third quartile not applicable) compared to patients in remission (*p* = 0.34). Patients in remission had the lowest MG-ADL-L scores (median = 0, first to third quartile of 0.0–1.0) compared to patients in IIa subgroup (*p* = 0.034).

**Table 3:** Latvian version of Myasthenia Gravis Activities of Daily Living (MG-ADL-L) score distribution according to myasthenia gravis severity.

MGFA class	Number of patients	MG-ADL-L median score (first to third quartile)
MGFA I	5	2.0 (0.5–7.0)
MGFA IIa	12	3.0 (1.3–3.0)
MGFA IIb	5	2.0 (1.0–7.5)
MGFA IIIb	3	5.0 (N/A)
Remission	13	0.0 (0.0–1.0)

MGFA, Myasthenia Gravis Foundation of America Classification.

## 4 Discussion

We translated, adapted and validated the MG-ADL for the Latvian population, generating the MG-ADL-L. There were no major difficulties during the translation process. We showed that MG-ADL-L is a reliable and valid tool to assess activities of daily living and functional impairment in Latvian patients with MG.

Based on Cronbach’s α (0.76), the MG-ADL-L is reliable. In other MG-ADL validation studies, the internal consistency has varied from 0.67 (Turkish version of the MG-ADL^12^) to 0.89 (Persian version of MG-ADL^9^). The different results and item-total-corrected correlations might be affected by the fact that Cronbach’s α increases if the items in the test are correlated to each other (14). The MG-ADL evaluates impairment of different types of activities, including eye function, the ability to brush teeth or comb hair, breathing, chewing and the ability to rise from a chair. These aspects can vary in each patient depending on MG type and dominant symptoms. There might not be a correlation between, for example, a patient’s eye function and the ability to his/her brush teeth and other aspects, so Cronbach’s α could be small and there could be weak item-total correlations.

The MG-ADL-L also had satisfactory reproducibility: there was acceptable agreement between the test and retest scores. Compared with other MG-ADL validation studies, which have used intraclass correlation coefficients (9–12), we used Cohen’s weighted kappa because it is more suitable for categorical data. All items of the MG-ADL-L showed moderate to near perfect agreement between the test and retest scores, implying excellent test–retest reliability, similarly to other MG-ADL validation studies (9–12).

We noted moderate construct validity, with positive MG-ADL-L associations with other scales used to evaluate patients with MG, namely the MGQOL15 and MGCS. Hence, the MG-ADL-L is a valid tool to assess impairment in daily activities in Latvian patients with MG.

We found a moderate correlation (r = 0.50) between the MG-ADL-L and the MGQOL15, similarly to validation study by Karanfil et al. (12), Faghani et al. (9) and Alanazy et al. (10) reported stronger correlations, but they used the revised MGQOL15 version, which could have affected the results.

The correlation between the MG-ADL-L and the MGCS was also moderate (r = 0.62). Faghani et al. (9) and Alanazy et al. (10) reported stronger correlations, an outcome that is expected given that the MGCS evaluates some of the same items, as they were derived from the MG-ADL.

Moderate correlation with the above-mentioned instruments could be explained by several impacting factors. The MG-ADL scale focusses on assessing the impact of myasthenia gravis (MG) on daily activities, capturing functional limitations experienced by people with MG. On the other hand, the MGQOL15 questionnaire assesses not only the functional domain but also the psychological and social domains, which could be affected even if activities of daily living are not impaired, therefore decreasing quality of life. Given this emphasis, it is expected that there would be moderate correlations between the MG-ADL-L scale and quality of life measures (such as MGQOL15). In this study, MGCS was evaluated by a physician (an objective measurement), but MG-ADL was completed by patients (a subjective measurement). We also observed an interesting discrepancy between objective symptoms, seen by physicians, and subjective symptoms, experienced by patients. In some cases, patients with seemingly no objective symptoms reported poor quality of life or showed worse MG-ADL scores than MGCS scores. This leads us to enquire about other factors that could affect the quality of life of patients with MG and their perception of their symptoms, for example, general fatigue, which is a very common symptom among patients with MG, but is not included on any of these scales; therefore, we have begun developing ideas for research on this topic.

There were differences in MG-ADL-L scores across the MG severity subgroups (as classified by the MGFA). Of note, the scores were similar in the MGFA I and IIB subgroups (both was a median MG-ADL-L score of 2). This result could be explained by the fact that there were only 5 patients in each of these subgroups. Including more patients in these subgroups could clarify whether there is actually a significant difference between them. Patients in remission showed the lowest MG-ADL score (median MG-ADL = 0), which is not surprising given that patients in remission experience fewer symptoms or none at all.

Our study had several limitations. First, we did not exclude patients for other common health-related problems and comorbidities, an approach that could have affected the outcome scores (e.g., Parkinson’s disease, joint pain, etc.). We noted the presence of other autoimmune diseases such as rheumatoid arthritis and autoimmune thyroiditis, which did not adversely affect the MG-ADL results compared to patients without such comorbidities.

Second, this was a single centre study, so there might be patient selection bias. It is important to note that due to a regional shortage of specialists in neuromuscular diseases like myasthenia gravis in Latvia, our centre serves not only local patients from Riga but also those from various other regions where there is a lack of specialized care. Given the rarity of myasthenia gravis and the specialized nature of its treatment, replicating our study across different centres in Latvia presents challenges due to the limited number of specialized facilities. Third, the distribution of patients across the MGFA groups was not balanced, as most of the participants were in remission or classified as MGFA IIA. However, it is important to mention that myasthenia gravis is characterised by variability in disease severity and fluctuation over time, with periods of remission or mild symptoms that alternate with exacerbations of varying severity. This variability in disease presentation can be challenging while achieving a perfectly balanced distribution across MGFA classes in study populations.

## 5 Conclusion

In conclusion, we translated and adapted the MG-ADL into the Latvian language according to standard translation and adaptation methods. We confirmed the reliability, reproducibility and validity of the resulting instrument, the MG-ADL-L. Therefore, this instrument can be used to evaluate the impact of MG on the daily activity of Latvian patients and to monitor the status of patients with MG during follow-up visits or clinical studies.

## Data availability statement

The raw data supporting the conclusions of this article will be made available by the authors upon a reasonable request.

## Ethics statement

The studies involving humans were approved by Riga Stradins University Ethics Committee (approval number: 2-PĒK-4/70/2023). The studies were conducted in accordance with the local legislation and institutional requirements. The participants provided their written informed consent to participate in this study.

## Author contributions

AG: Data curation, Formal analysis, Writing – original draft. IR: Data curation, Investigation, Writing – review & editing. MR: Investigation, Writing – review & editing. GĶ: Investigation, Writing – review & editing. VŽ: Investigation, Writing – review & editing. IG: Investigation, Writing – review & editing. MZ: Formal analysis, Methodology, Writing – review & editing. VK: Conceptualization, Data curation, Investigation, Supervision, Writing – review & editing.
